# Toxigenic and non-toxigenic patterns I, II and III and biofilm-forming ability in *Bacteroides fragilis* strains isolated from patients diagnosed with colorectal cancer

**DOI:** 10.1186/s13099-020-00366-5

**Published:** 2020-06-05

**Authors:** Seyedesomaye Jasemi, Mohammad Emaneini, Mohammad Sadegh Fazeli, Zahra Ahmadinejad, Bizhan Nomanpour, Fatemah Sadeghpour Heravi, Leonardo A. Sechi, Mohammad Mehdi Feizabadi

**Affiliations:** 1 Department of Microbiology, School of Medicine, Tehran University of Medical Sciences, Tehran, Iran; 2 Department of Surgery, Imam Khomeini Hospital Complex, Tehran University of Medical Sciences, Tehran, Iran; 3 Department of Infectious Diseases, Imam Khomeini Hospital Complex, Tehran University of Medical Sciences, Tehran, Iran; 4 Department of Microbiology, School of Medicine, Kermanshah University of Medical Sciences, Kermanshah, Iran; 5 Surgical Infection Research Group, Faculty of Medicine and Health Sciences, Macquarie University, Sydney, Australia; 6 Department of Biomedical Sciences, University of Sassari, Sassari, Italy

**Keywords:** Enterotoxigenic *Bacteroides fragilis*, ETBF, *bft* gene, Biofilm, Colorectal cancer, CRC

## Abstract

**Background:**

*Enterotoxigenic Bacteroides fragilis* (ETBF) associated with the initiation and progression of colorectal cancer (CRC) has been alarmingly reported all over the world. In this study, simultaneous investigation of toxigenic and non-toxigenic patterns I, II and III and biofilm formation ability of *Bacteroides fragilis* isolated from patients with colorectal cancer was performed.

**Methods:**

Thirty-one patients diagnosed with CRC and thirty-one control subjects were recruited in this study. Specimens were cultured on BBE and BBA culture media. Classical phenotypic identification tests and PCR was performed to verify *Bacteroides fragilis* presence. Also, biofilm-forming ability and expression of *bft* gene were assessed under biofilm and planktonic forms.

**Results:**

A total of 68 *B.fragilis* was isolated from all colorectal tissue, of which 13 isolates (19.1%) (11 isolates from CRC and 2 from normal tissue) were positive for *bft* gene. The abundance patterns of I, II and III were as follow in descending order; pattern I > pattern III > pattern II in CRC subjects and pattern II > pattern III > pattern I in normal tissues. Also, pattern I showed higher biofilm formation ability compared to other patterns. Toxin expression was significantly reduced in biofilm form comparing with planktonic form.

**Conclusions:**

Based on our findings, there was a difference between the abundance of patterns I, II, and III and biofilm formation in isolates obtained from CRC and normal tissues. Biofilm formation ability and toxin encoding gene (*bft*) are two main virulence factors in *B. fragilis* pathogenicity which require more investigation to treat *B. fragilis* infections effectively.

## Background

Colorectal cancer (CRC) is the third common cancer in the world and the second leading cause of cancer-related deaths in 2018 [1]. Several genetic and environmental factors are implicated in CRC development [1]. Enterotoxigenic *Bacteroides fragilis* (ETBF) is the most common carcinogenic bacteria and one of the main environmental factors involved in CRC development [1–3].

After the attachment of *Bacteroides fragilis* toxin to corresponding receptors, in an ATP-dependent process, cell membrane proteins such as E-cadherin protein is stimulated. Activation of β-catenin and NFκB signaling pathways leads to the initiation of proinflammatory signals. As a result, during a process known as epithelial-mesenchymal transition (EMT), epithelial cells lose their epithelial functions including cell–cell interactions and cell polarity and lead to the metastatic phenotype [4, 5]. By raising the reactive oxygen species (ROS) level, this toxin can also affect the DNA host as well [6].

*B. fragilis* toxin (BFT) is coded by the *bft* gene with three isotypes, namely *bft*-*1*, *bft*-*2*, *bft*-*3*, located on a pathogenicity island (PAI). The PAI enters bacteria’s chromosome from the flanking region (conjugative transposon CTn86). Nontoxigenic *Bacteroides fragilis* (NTBF) strains do not possess a PAI, but the presence of flanking region in certain strains allows the PAI to pass from ETBF to NTBF strains [7, 8].

Based on this assumption, three patterns are assigned to *Bacteroides fragilis*. Enterotoxigenic *B. fragilis* strains (ETBF) with *bft* gene is defined as pattern I and non-toxigenic strains (NTBF) are defined as Pattern II and III. Pattern II defines as strains without the pathogenicity island region and flanking region and pattern III defines as strains without the pathogenicity island and with flanking region [9] Various studies have shown, ETBF (*Bacteroides fragilis* belong to pattern I) increased in cancer colorectal samples compared to healthy individuals [10, 11].

At the same time, studies suggest that biofilm formation by *B. fragilis* is closely related to CRC, and *B. fragilis* in biofilms can be a diffusion barrier that causes antibiotic access limitation and survive in hostile environments [12]. In other words, the bacterial–bacterial and host-bacterial interactions taking place in the biofilm can also affect the intestinal epithelium metabolism and lead to excessive cellular proliferation and CRC initiation and progression [13, 14].

Due to the importance of toxin and biofilm formation in pathogenicity of *Bacteroides fragilis* and development of CRC, we have investigated the profile patterns of *bft* gene i.e. I, II, and III and biofilm formation ability in *Bacteroides fragilis* isolated from colorectal cancer (CRC) tissues in this study.

## Results

### Patient population

In this case–control study, 62 biopsy samples were collected from patients and healthy individuals referring to colonoscopy Unit of Tehran’s Imam Khomeini Hospital. 31 (50%) biopsy samples were extracted from CRC tissue and 31 (50%) from normal colorectal tissue. Patient demographics is presented in Table 1.

**Table 1:** Characteristics of Patient population and controls

Patient characteristic	CRC N (%) 31 (50)	NC N (%) 31 (50)
Age median	58	58
Mean ± SD	59.03 ± 11.18	57.35 ± 10.79
Gender n (%)		
Female	13 (41.9)	16 (51.6)
Male	18 (58.1)	15 (48.4)

*CRC* Colorectal cancer tissue, *NC* Normal Colorectal tissue, *N* Number of patients

### *Bacteroides fragilis* isolation

A total of 82 suspicious isolates to *Bacteroides fragilis* were isolated from 35 biopsy samples (56.5%). Phenotypic and PCR identification of *Bacteroides fragilis* were validated using specific primers of 16S rRNA gene region. From the 82 suspicious isolates, 68 were *Bacteroides fragilis* (Table 2). GenBank accession numbers for 16S rRNA gene sequencing is: MN955555.1, MN955554.1, MN950426.1, and MN937242.1.

**Table 2:** *Bacteroides fragilis* isolated from CRC and NC and their patterns

Biopsy N (%)	*B.fragilis* N (%)	*bft* gene N (%)	PatternI	PatternIIN	PatternIII	Biofilm formation		
						Weak	Moderate N	Strong
NC biopsy 31 (50)	32 (47.1)	2 (6.25)	2	23	12	5	18	9
CRC biopsy 31 (50)	36 (52.9)	11 (30.5)	11	8	12	0	18	18
62 (100)	68 (100)	13 (19.1)	13 (19.1)	31 (45.6)	24 (35.3)	5 (7.4)	36 (52.9)	27 (39.7)

*CRC* Colorectal cancer, *NC* Normal Colorectal tissue, *N* Number of patients

### Identification of *bft* gene and patterns I, II, and III

*bft* gene and its isotypes was validated with PCR using specific primers for the gene region. GenBank accession numbers for *bft* gene is MK792343.1. The presence of this gene in CRC was significantly higher than normal tissue (*P *= 0.011). Thirteen isolates (19.1%) had *bft* gene (11 extracted from CRC tissue and 2 from normal colorectal tissue). PCR isotype determination revealed the presence of *bft*-*1* in 12 isolates and *bft*-*2* in a one isolate. Isotype *bft*-*3* was not identified.

Pattern I ETBF was detected in 13 isolates. Twenty-four NTBF isolates were flanking region-PCR positive, suggesting pattern III in these strains, and 31 were flanking region-PCR negative, indicating pattern II in these strains. Table 2 displays the abundance of patterns for the isolated strains of *Bacteroides fragilis* extracted from CRC and normal colorectal tissues.

### Biofilm formation

**Fig. 1:**
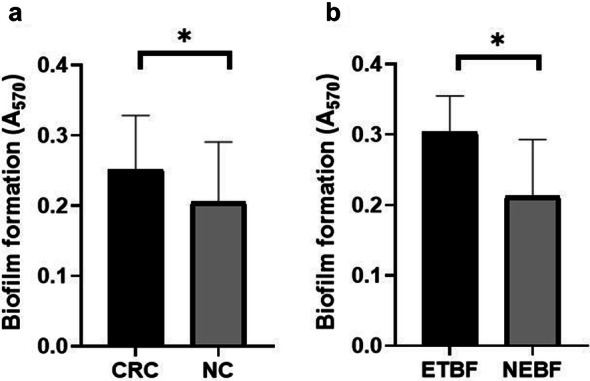
**a** Biofilms of *B. fragilis* strains were stained with 1% crystal violet and evaluated by measuring the absorbance at A570. The black bars represent the average ± SD (0.25 ± 0.07) of in *B. fragilis* strains isolated from CRC and gray bars represent the average ± SD (0.20 ± 0.08) of in *B. fragilis* strains isolated from NC. *Indicates statistical significance (*P *= 0.022). **b** The black bars represent the average ± SD (0.30 ± 0.05) of ETBF strains and gray bars represent the average ± SD (0.21 ± 0.08) of in NEBF strains. *Indicates statistical significance (*P *= 0.001)

Biofilm formation was monitored by means of OD measurements of individual strains. According to the classification of the isolates based on the ability to adhere to the base of the wells and produce biofilm, 5 isolates (7.4%) showed “weak” (+ 1), 36 isolates (52.9%) exhibited “moderate” (+ 2), and 27 isolates (39.7%) showed “strong” (+ 3) biofilm-forming ability. All *Bacteroides fragilis* isolates derived from CRC tissue possessed a medium to strong biofilm-producing ability. The ability of biofilm formation in strains isolated from colorectal cancer and normal tissues is shown in Table 2. There was a meaningful difference in the ability of biofilm formation of CRC-extracted *Bacteroides fragilis* isolates as compared to those derived from normal tissue (*P *= 0.022) (Fig. 1a). Also, ETBF strains had significantly higher ability to biofilm formation than NEBF strains (*P *= 0.001) (Fig. 1b).

### *bft* gene expression under biofilm and planktonic conditions

**Fig. 2:**
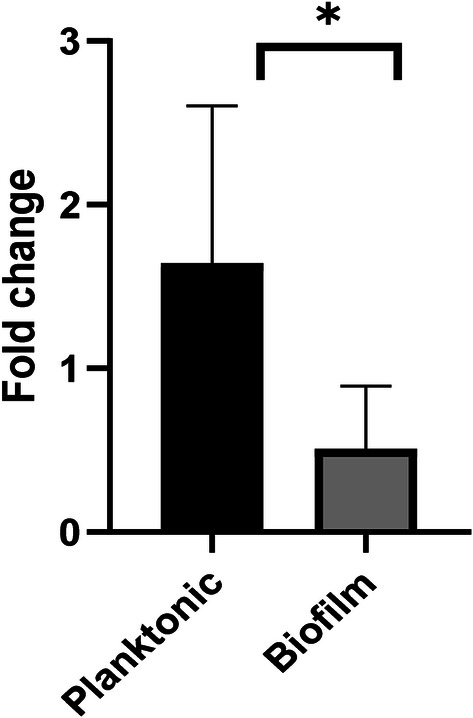
Comparison of *bft* gene expression in *Bacteroides fragilis* strains under planktonic and biofilm conditions. The black bars represent the average fold-change ± SD (1.64 ± 0.96) of *bft* gene expression under planktonic condition and gray bars represent the average fold-change ± SD (0.50 ± 0.38) of *bft* gene expression under biofilm condition. *Indicates statistical significance (*P *= 0.001)

Real-time PCR quantification of *bft* gene expression under biofilm and planktonic conditions was conducted and the fold change calculated. The relative expression level of *bft* gene was 3.28-fold higher in planktonic cells than in biofilm growth of *B.fragilis* strains which was statistically significant (*P* = 0.001) and shown in Fig. 2.

## Discussion

In the present study, we have investigated the profile patterns I, II, and III and the biofilm-forming ability in CRC-extracted *Bacteroides fragilis* isolates and normal tissue. To best of our knowledge it was the first study to evaluate biofilm-forming ability and toxin expression of CRC-extracted *Bacteroides fragilis* isolates under planktonic and biofilm conditions in our region.

The study suggests a meaningful difference between the presence of *bft* gene in CRC-derived isolates compared to isolated strains from normal tissue (*P *= 0.011). Several studies have also supported the existence of a meaningful relation between the presence of *bft* gene and CRC [3, 10, 11]. Few studies have conducted in Iran to study the relationship between ETBF and CRC [15]. For instance, Haghi et al. examined 60 faeces samples in patients diagnosed with CRC and 60 faeces samples in healthy individuals to identify ETBF via direct PCR. ETBF strains were detected with higher frequency among CRC patient than healthy control.

The relationship between *Bacteroides fragilis* and CRC has been studied in other parts of the world. In the study conducted by Boleiji et al. all “stage III” (severe infection) CRC samples were *bft*-positive [16]. The prevalence of “stage I” and “stage II” was 72%. Topark et al. (2006) also demonstrated a meaningful difference in *bft* gene presence in faeces samples of CRC patience compared to normal individuals (38% and 12% respectively) [11].

*bft* gene has three isotype variants, pathologically expressed as *bft*-*2 *> *bft*-*1 *> *bft*-*3*. In the present study, ETBF strains were examined in terms of isotype toxin. Twelve isolates possessed *bft*-*1* and in a single isolate the presence of *bft*-*2* was confirmed. None of the isolates exhibited *bft*-*3*. Based on previous findings in various geographic regions such as Iran, Turkey, and the USA *bft*-1 was the most common isotype toxin [17–19].

*Bacteroides fragilis* isolates are classified in three patterns based on *bft* gene presence (PAI) and its flanking region. The presence of all three patterns were investigated in this study. The abundance of patterns detected in CRC-extracted isolates was as follow pattern I > pattern III > pattern II compared to II > III > I for isolates extracted from normal tissue.

The majority of CRC-derived *Bacteroides fragilis* isolates exhibited pattern I (ETBF strains). In contrast, this pattern was the least common pattern detected in healthy individuals. These findings underline the significant role of PAI and its flanking region in CRC pathogenesis and their correlation with this disease.

In the majority of isolates collected from normal tissue, *Bacteroides fragilis* isolates exhibited pattern II which lack PAI and flanking region. Pattern III (strains lacking PAI but possessing flanking region) was observed in CRC-extracted NTBF isolates in 11 isolates and from 8 isolates from normal tissues. Phylogenic studies suggest the possibility of PAI transfer to other isolates and subsequent transformation to ETBF over time [7]. Meanwhile, no study was reported to evaluate the distribution of these patterns in CRC-extracted isolates in Iran.

In a study conducted by Claros et al. 63 *Bacteroides fragilis* isolates extracted from blood and 197 isolates derived from other clinical samples were investigated. In blood samples, 43%, 38%, and 19% of isolates exhibited patterns II, III and I respectively. In other clinical samples, the frequency of patterns II, III and I was as follow the result was 47%, 43%, and 10% respectively which is similar to the patterns discovered in our study in normal tissues [20]. Different studies have revealed a direct correlation between ETBF (pattern I) and CRC development. In other words, the presence of *bft* gene (pattern I) has been associated with CRC development [10, 11, 15].

Concurrently, the biofilm-forming ability of *Bacteroides fragilis* was detected by staining the bacteria attached to the base of the microplate with crystal violet dye. Results indicated a high biofilm formation ability in ETBF strains compared to NTBF, which was statistically meaningful (*P *= 0.001). In the study conducted by Pierce et al. several NCTC strains were examined [21]. They showed that toxin-generating strains were more capable of biofilm formation compared to non-toxin generating strains. Biofilm-forming ability is a crucial feature of bacteria which is involved in antibiotic resistance, ETBF colonization, adherence to the epithelial surface, and prevention of toxin dissemination.

In the present study, strains isolated from CRC tissue showed higher biofilm-forming ability compared to isolates of normal tissue, which was also a statistically meaningful finding (P = 0.022). Based on the obtained results, biofilm-forming ability, with or without toxin, may be associated with CRC development. Studies have also demonstrated the effect of multi-bacterial biofilms on the increase of polyamine metabolites which may intensify CRC growth, invasion, and metastasis [22].

Changes in *bft* gene expression under planktonic and biofilm growth was also considered in this study. The *bft* expression showed a statistically meaningful reduction under biofilm condition. Meantime, no studies were found to compare *bft* gene expression under biofilm and planktonic conditions so far.

The findings here possibly suggest that *bft* gene has no significant role in the biofilm formation process. Similarly, other studies also show that the absence of *bft* gene in non-toxin generating strains does not reduce biofilm formation, which suggests that toxin may not be a crucial factor for the formation of this phenotype [22]. Studies have also identified the impact of toxin regulating two-component system RprXY on *bft*-gene expression in vivo and in vitro [23]. This system regulates the expression of *bft*-gene. Although, 30% of *Bacteroides fragilis* in the gastrointestinal system have *bft* gene, it is in suppressed state [24]. *Bft* gene expression may increase depending on dynamic interaction between intestinal mucosa with toxin and the two-component system. Hence, further investigation in vivo and in vitro is required to quantify *bft*-gene expression in the CRC tissues under biofilm condition. In addition, evaluation of biofilm formation ability and toxin expression of strains isolated from different stage of cancer (I, II, III and IV) is suggested.

### Conclusions

In the present study, pattern I, II, and III profiles among *Bacteroides fragilis* isolates was different from isolates obtained from CRC patients and normal individuals. Pattern I was the most common pattern in CRC isolates and exhibited greater biofilm-forming ability compared to patterns II and III.

These findings suggest a possible correlation between *bft* gene presence and biofilm-forming ability in *Bacteroides fragilis* and CRC development. However, further studies are needed to evaluate the role of pattern I and biofilm in the development of CRC and to target toxin-expression and bacterial biofilm more effectively as an efficient strategy in the treatment of colorectal cancer.

## Methods

### Patient population

In this study, 31 patients with the mean age of 59.03 (SD = 11.18) with a clinically diagnosed CRC confirmed by radiographic, pathologic, and colonoscopy examination were enrolled. Patients had not received any antibiotic treatment for one month prior to the experiment. Thirty-one healthy individuals with age matched control (Mean ± SD = 57.35 ± 10.79) whit no intentional disorders were also recruited in this study.

### Sample collection

Biopsy samples were obtained from each participant over 9 months by a gastroenterologist (August 2018 to April 2019). Samples were taken from the colon and rectum. 80.6% of tumor samples was in stage II. Samples were placed to thioglycolate broth (THIO) transport medium containing vitamin K1 (0.5 mg/l) and hemin (5 mg/l) for 30 s before being sent to the microbiology laboratory.

### Samples preparation and isolation of *Bacteroides fragilis*

Samples were homogenized using a mortar and pestle upon arrival at the lab. 2 to 3 drops of homogenized samples (without delay) was transferred to inoculate Bacteroides Bile Esculin Agar (BBE) and Brucella Blood Agar (BBA) containing 5% sheep blood, 0.5 mg/l vitamin K1, and 5 mg/l hemin and incubated for 48–72 h at 37 °C under anaerobic condition. 5–10 grey colonies grown on BBE and BBA were re-cultured in a BBA medium. Aerotolerance test was conducted to ensure that the target bacteria was absolutely anaerobic. Anaerobic coccobacilli with positive bile esculin and negative catalase tests were transferred to BHI broth containing 15% glycerol and stored at − 80 °C.

### Bacterial species identification

To determine type and species of *Bacteroides fragilis*, two polymerase chain reactions was used for amplification of 16S rRNA gene region. First reaction was reserved for verifying *Bacteroides fragilis* group and the second for determining its species [25, 26]. PCR primers and product size are presented in Table 3. The 16S rRNA gene was sequenced for some strains and submitted in GenBank.

**Table 3:** The primers used in this study

Target region	Sequence 5′ to 3′	Amplicon size (bp)
*B fragilis* group	F: ATAGCCTTTCGAAAGRAAGAT R: CCAGTATCAACTGCAATTTTA	495
*B fragilis*	F: TCRGGAAGAAAGCTTGCT R: CATCCTTTACCGGAATCCT	163
All *bft* gene	F: GGATACATCAGCTGGGTTGTAG R: GCGAACTCGGTTTATGCAGT	296
*bft*-1	F: TCTTTTGAATTATCCGTATGCTC R: CTTGGGATAATAAAATCTTAGGGATG	169
*bft*-2	F: ATTTTTAGCGATTCTATACATGTTCTC R: GGGCATATATTGGGTGCTAGG	114
*bft*-3	F: TGGATCATCCGCATGGTTA R: TTTGGGCATATCTTGGCTCA	148
Flanking region	F: TTCAACCTGATCGATCCGGAAGATCCG R: GCTGGTAGACTACCTGAGTAAGGAGTC	1600
BFT qRT-PCR	F: AAGGGCTGGATGGCTTTACT R: GGGATACATCAGCTGGGTTG	–
16S qRT-PCR	F: CAGTCTTGAGTACAGTAGAGGTGG R: GTGGACTACCAGGGTATCTAATCC	–

### Identification of *bft* gene and its isotypes

PCR was used to detect *bft* gene and the isotypes as previously described by Odamaki T et al. [27]. Primer properties is presented in Table 3.

### Identification of patterns I, II, and III

Based on the evidence provided by previous studies [8], ETBF strains exhibit pattern I. Hence, pattern I consists of strains of *Bacteroides fragilis* that possess *bft* gene. NEBF strains exhibit patterns II and III. To separate pattern II from pattern III, selected sections of the flanking region was amplified by PCR. Primer properties is presented in Table 3.

### Biofilm formation

Microtiter plate assay was used to investigate biofilm formation ability in vitro [28]. Briefly, several colonies from the fresh culture were diluted in BHIS broth to obtain a microbial suspension with a concentration of OD = 0.08–0.1. Once done, 20 μl of the microbial suspension was added to a microplate containing 180 μl of BHIS broth. The same procedure was repeated for each strain in three separate wells, and cultures were incubated for 24 h at 37 °C under anaerobic condition. At the subsequent stage, the upper layer medium of wells was disposed, and the wells were rinsed with 100 ml of PBS (pH = 7.2). Microplates were left for 10 min at 65 °C to dry. Then, 200 µl of crystal violet (1%) was added and incubated for 5 min at room temperature. To dissolve the colour attached to biofilm, 150 µl of acetic acid at 30% concentration was added to each well and absorbed crystal violet was measured at 570 nm by an ELISA reader.

### *Bft* gene expression under planktonic and biofilm conditions

To determine *bft*-gene expression under planktonic condition, *Bacteroides fragilis* strains were cultured in Brucella Blood agar (BBA) and incubated for 24 h at 37 °C. Then, several colonies from the fresh culture were diluted in BHIS broth and incubated for 16 h at 37 °C under anaerobic condition.

To examine *bft* expression under biofilm condition, several colonies from the fresh culture were diluted in BHIS broth to obtain a microbial suspension with a concentration of OD = 0.8–0.1. Once done, 20 μl of the microbial suspension was added to a microplate containing 180 μl of BHIS broth and incubated for 24 h at 37 °C under anaerobic condition. At the subsequent stage, the upper layer medium of wells was disposed, and the wells were rinsed with 100 ml of PBS (pH = 7.2). Using a sterile pipette and pipette tip, cells were scraped off the base and walls of the well, diluted in 100 μl of PBS, and collected.

### RNA extraction

RNA was extracted subsequently using an RNX-Plus solution kit (Sinaclon-Iran) according to the instruction protocol. DNase I, RNase free kit (Sinaclon-Iran) was used to avoid possible genomic DNA contamination. Finally, cDNA was synthesized using a Reverse Transcription kit (Sinaclon-Iran) with random hexamer primers.

### Real-time PCR

Expression of *bft* gene was quantified by specific primers and SYBR Green real-time PCR [29]. Primer properties is presented in Table 2. PCR conditions included an initial denaturation at 95 °C for 5 min, followed by a 40-cycle amplification consisting of denaturation at 95 °C for 20 s and annealing and extension at 59 °C for 30. Specificity of PCR reactions was verified by melt graph analysis. Gene expression level was normalized by 16S rRNA sequence, and gene expression was quantified by ΔΔCT method.

### Statistical analysis

Data were analyzed using the SPSS ver. 18.0 (SPSS Inc., Chicago, IL). The Chi square test was used to analyze the data on the presence of ETBF and NTBF strains, CRC tissue in comparison to normal tissue. Independent *t* test were performed to analyze the data for biofilm formation in *Bacteroides fragilis* isolated from CRC tissue as compared to the normal tissue. Also, significant difference in biofilm formation between ETBF and NETB was calculated by Independent t-test. Pairwise Student’s t- test was performed to analyze the data for *bft* gene expression in planktonic phase and biofilm phase. A P-value < 0.05 was considered as statistically significant.

## Abbreviations

CRC: Colorectal cancer
ETBF: Enterotoxigenic *Bacteroides fragilis*
NTBF: Nontoxigenic *Bacteroides fragilis*
